# Quality of multiple sclerosis out-patient health care services with focus on patient reported experiences

**DOI:** 10.1186/s13104-017-2568-y

**Published:** 2017-07-06

**Authors:** Anne Marit Solheim, Åse Mygland, Unn Ljøstad

**Affiliations:** 1 Department of Neurology, Sørlandet Hospital Kristiansand, Service Box 416, 4604 Kristiansand, Norway; 2 Department of Clinical Medicine, University of Bergen, Bergen, Norway

## Abstract

**Background:**

To investigate multiple sclerosis (MS) patients’ satisfaction with out-patient follow-up in a general neurological hospital department. Patients with definite MS living in Vest-Agder county, Norway were invited to answer a questionnaire comprising one question regarding overall satisfaction, and 24 questions regarding demographics, disease characteristics, and experiences with different aspects of the health care services.

**Results:**

Out of 330 invited patients, 159 responded (48%). Mean overall satisfaction with health care was 3.5 (SD = 1.03) on a 1–5 Likert scale (1 = not at all, 5 = to a very large extent). The best sub scores were given on confidence in the physician’s competence (mean = 4.01), the physician speaks in an understandable way (mean = 4.07), expectation of good treatment (mean = 3.72), and perception of being submitted to wrong treatment (mean = 1.5). The worst scores were given on satisfaction with frequency of outpatient appointments (mean = 2.89) and delay of outpatient appointments (mean = 3.07). Four factors were associated with high overall satisfaction; receiving the disease modifying drug natalizumab (B = 0.549, p = 0.004), satisfaction with frequency of outpatient appointments (B = 0.242, p < 0.001), experience that the physician facilitates talking about what the patient finds important (B = 0.218, p = 0.001), and confidence with the physician’s competence (B = 0.453, p < 0.001).

**Conclusion:**

The patients were rather satisfied with the content of follow-up, and less satisfied with the structure. Regular and predictable contact with a trustworthy physician that facilitates that the patient is able to talk about what is important was associated with higher overall satisfaction.

**Electronic supplementary material:**

The online version of this article (doi:10.1186/s13104-017-2568-y) contains supplementary material, which is available to authorized users.

## Background

Multiple sclerosis (MS) is a chronic and lifelong disease that may cause disability and impair quality of life. Disease course and treatment vary substantially among MS patients [1], and the follow-up require an individual approach within the framework of a comprehensive and high quality health care service program.

Benchmarking of health care quality have traditionally focused on complication rates, re-admission rates, 30-days mortality rate, breaches of waiting lists and other clinical performances. During recent years, however, there has been growing awareness of patient satisfaction as a major quality measure [2]. The importance of recognition and incorporation of Patient Reported Experience Measures (PREMs) in assessment of health care quality is grounded on a positive relation between patient satisfaction and clinical outcomes and safety [3–9]. Further, it is documented that patient satisfaction affects medical malpractice claims, and personal and professional satisfaction [9].

Norwegian national guidelines for MS care [10] recommend regular and predictable follow-up for all MS patients, regardless of disease severity and course. We have little knowledge about the extent to which these guidelines are followed in everyday clinical practice, and we have even less knowledge about patients’ satisfaction with the different aspects of MS care. The aim of this study was to assess quality of MS healthcare services with focus on PREMs and satisfaction with content and structure of out-patient care in patients with MS at Sørlandet Hospital Kristiansand, Norway.

## Methods

We identified all patients in the Norwegian county Vest-Agder diagnosed with MS between 1996 and 2010 according to Poser or McDonald criteria by search in medical records. During the period 2012–2013 the patients received a letter with an invitation to participate in the study. They were asked to return the enclosed questionnaire in a completed state by prepaid mail. The answers were anonymous. We sent no reminders. Due to anonymity we could not identify and record data on non-responders.

Sørlandet Hospital in Kristiansand city has the only neurological ward in Vest-Agder, a county with a population of approximately 170,377 in 2010, a catchment area of 7,276,51 km^2^, and an estimated MS prevalence of 180 per 100,000 population [11]. There is one private neurologist in the area with practice for MS patients. Patients who had moved out of the county or received follow-up outside Sørlandet Hospital Kristiansand were not included in the analysis.

The MS care at Sørlandet Hospital Kristiansand is organized in a general out-patient clinic with neurologists and neurology trainees. The out-patient clinic has a MS nurse, but is otherwise not specialized for MS care. The appointments with physicians last 45 min, and are in general scheduled at least every 12 months. There may be delays in scheduled appointments by up to 6 months. Patients receiving treatment with natalizumab have the drug administered every fourth week by nurses with special knowledge of MS treatment, and they have a physician appointment every 6 months.

We constructed a questionnaire (Additional file 1) based on the principles of “PasOpp”, a validated questionnaire for evaluating somatic out-patient clinics in Norway [4], and on a previous Norwegian study on patients’ satisfaction [12]. Our questionnaire comprised nine questions covering demographics, disease characteristics, mobility and treatment, and 16 questions regarding expectations, structure and content of follow-up. To assess overall patient satisfaction we asked “*How satisfied are you with the help you have received for your MS disease at Sørlandet Hospital Kristiansand*?” All answers, except those regarding demographic data, disease characteristics, knowledge of contact physician, frequency of out-patient appointments, and whether the patients had visited the MS nurse were given as a score on a 5 point Likert scale, ranging from 1 being “not at all” to 5 being “to a very large extent”. Likert scaling is widely used by researchers, also in studies evaluating patient satisfaction [13], as the range of positive or negative responses to a statement may be easily understood and communicated by the respondent.

### Statistics

**Fig. 1:**
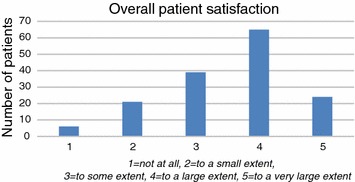
Overall satisfaction with healthcare services. Total number 155. Mean 3.5 (SD = 1.03)

We consider the Likert scale to be quite symmetric with equidistant attributes and therefore a defensible approximation to an interval scale. Consequently, the results are reported as mean scores with standard deviation. For the same reason we used a linear regression model for analysis of association between different variables and overall patient satisfaction. The dependent variable in this analysis was overall satisfaction (Fig. 1), and independent variables were demographic data, disease characteristics and PREMs (Tables 1, 2). Variables with p values <0.05 in the univariate analyses and with less than 10 missing data were entered into a stepwise multivariate analysis using a general linear model together with the question “*Have you visited the MS* nurse?” (p = 0.084). Variables with p values <0.05 were considered significant in the multivariate analyses.

**Table 1:** Demographic data, disease characteristics and patients experience with follow-up. Relapsing remitting MS (RR MS), secondary progressive MS (SP MS), primary progressive MS (PP MS)

Demographics	
Age (n = 159) (years) [mean (SD)]	54 (11.5)
Sex (n = 158) M/F [N (%)]	39/119 (25/75)
Education, years after primary school (n = 156) <3/3–6/>6 [N (%)]	62/62/32 (39/39/20)
Employment status (n = 153)	
Full time/part time/disabled or retired [N (%)]	29/20/104 (19/13/68)
Marital status (n = 155)	
Single or widowed/married or cohabitant [N (%)]	30/125 (20/80)

Disease characteristics	
Type of MS (n = 159)	
RR/SP/PP/Unknown [N (%)]	71/22/17/49 (45/14/11/31)
Duration of MS (n = 157) (years) [mean (SD)]	12 (9.1)
Walking distance without support (n = 153)	
Unlimited/>100 m/<100 m [N (%)]	66/35/52 (41/22/33)
Use of mobility aids in daily life (n = 148)	
None/walker, cane or other/wheelchair [N (%)]	84/28/36 (53/18/23)
MS treatment now (n = 156)	
None/Interferones or glatirameracetate/natalizumab/fingolimod [N (%)]	104/35/14/6 (65/22/9/4)

**Table 2:** Patient reported experiences of the out-patient physician follow up at Sørlandet Hospital Kristiansand

Questions	Number of patient reports on a 1–5 Likert scale^a^					Mean (SD)
	1	2	3	4	5	
Expectations						
Did you have expectations of receiving good treatment at the hospital when diagnosed with MS? (n = 158)	5	9	36	83	25	3.72 (0.91)
Structure						
How much do you consult your GP about your illness? (n = 159)	23	82	41	12	1	2.28 (0.83)
Have you seen many different physicians in the outpatient clinic? (n = 149)	40	36	39	13	21	2.59 (1.5)
Do you think the outpatient appointments are often enough? (n = 152)	31	28	36	40	17	2.89 (1.31)
Does it happen that your outpatient appointments are later than previously agreed upon? (n = 144)	28	20	39	28	29	3.07 (1.4)
Are you satisfied with the availability of the physician? (n = 141)	13	18	45	48	17	3.27 (1.12)
Do you think the outpatient appointments last long enough? (n = 145)	8	14	35	63	25	3.57 (1.06)
Content						
Have you received adequate information about your disease and treatment options? (n = 153)	18	22	43	43	27	3.25 (1.24)
Do think the physicians facilitate that you can talk about what is important to you? (n = 152)	16	11	35	58	32	3.52 (1.20)
Are you involved in decisions involving your treatment?	11	19	27	59	31	3.54 (1.18)
Do you trust the physician’s professional competence? (n = 153)	0	8	29	70	46	4.01 (0.84)
Do the physicians speak to you in an understandable way? (n = 154)	4	5	18	76	51	4.07 (0.90)
In your opinion, have you been submitted to wrong treatment in any way? (n = 145)	101	24	14	4	2	1.50 (0.88)

^a^ Reported on a 1–5 Likert scale: 1 = not at all, 2 = to a small extent, 3 = to some extent, 4 = to a large extent, 5 = to a very large extent

A statistical software package for analyses (SPSS, version 21) was used.

## Results

Out of 330 invited patients, 159 answered the questionnaire (Additional file 2) (response rate 48%). The overall patient satisfaction given as a score from 1 to 5, where 1 is “not at all” and 5 is “to a very large extent” is shown in Fig. 1. The mean score was 3.5 (SD = 1.03). A proportion of 89 out of 155 (57%) patients scored 4 (“to a large extent”) or 5 (“to a very large extent”) on the question *“How satisfied are you with the help you have received for your MS disease at Sørlandet Hospital, Kristiansand*”.

Demographics and disease characteristics are shown in Table 1. Questions and scores regarding expectations, structure and content of follow-up are shown in Table 2. Three additional questions were *“do you know if you have a contact physician?”*, *“how frequent are your outpatient appointments?”*, and *“have you visited the MS nurse?”* 74% reported that they knew their contact physicians, 50% reported the frequency of outpatient contact as at least once per year, and 60% reported that they had visited the MS nurse.

In univariate analyses the following factors had a significant association with overall satisfaction: age (p = 0.040), type of MS (p = 0.016), current MS treatment (p = 0.016), level of handicap (walking distance p = 0.010 and use of mobility aids p = 0.002), knowledge of contact doctor (p < 0.001), frequency of contact (p = 0.001), and variables concerning patient reported experiences and satisfaction. In multivariate analysis, using a general linear model, four factors remained significantly associated with overall satisfaction; receiving the disease modifying drug natalizumab, satisfaction with frequency of outpatient appointments, experience that the physician facilitates talking about what’s important for the patient, and confidence with the physician’s competence (Table 3).

**Table 3:** Variables positively associated with overall satisfaction in multivariate analysis

	B	p value
Receiving disease-modifying treatment		
Interferones/glatirameracetate	0.176	0.153
Natalizumab	0.549	0.004
Fingolimod	0.381	0.139
Assessment of appointments being often enough	0.242	<0.001
Confidence in physician’s competence	0.453	<0.001
Being able to talk about what is important	0.218	0.001

R squared = 0.673

## Discussion

The study results indicate a rather high overall patient satisfaction among our MS patients, as almost 60% scored “to a large extent” or “to a very large extent” on the question *“How satisfied are you with the help you have received for your MS disease at Sørlandet Hospital, Kristiansand*”. The patients were also rather satisfied with the content of the follow-up, as the majority scored “to a large extent” or “to a very large extent” on trusting the physician’s competence, satisfaction with communication, and involvement in decision making. Among the variables evaluating content issues, the patients were least satisfied with the offered information. This underlines that continuous and customized information is fundamental. Previous studies have also highlighted the importance of satisfactory information being offered MS patients [1, 14].

The scores on questions regarding the structure and frequency of follow-up were generally lower than scores questions regarding content. According to Norwegian recommendations [2], MS patients should be assigned a contact physician, be offered at least annual follow-up, and have easy access to a contact physician between planned visits if required. Our results show that the out-patient follow-up of MS patients at Sørlandet Hospital, Kristiansand is neither as structured nor coordinated as guidelines recommend, and not as accessible as the patients wants. Interestingly, a Swedish study found almost similarly that only 65% of MS patients were satisfied with the accessibility of doctors, but as many as 80–90% were satisfied with engagement and treatment provided by the physician [15]. The identified suboptimal organization of our health care services should be met and efforts provided accordingly. Solutions to be considered may be to ensure allocation of a contact physician to every MS patient, to increase MS nurse services [16], to develop open rapid-access services [17] and to improve the interaction between primary and secondary care. In this context it is noteworthy that as many as 14% of our patients did not consult their GP at all about their MS, and 52% did so but only to a small extent. In a qualitative study from 2003 of care coordination, many patients reported that lack of communication between providers involved in their care was an obstacle to coordinated care [18].

Four variables were positively associated with overall satisfaction namely receiving the disease modifying drug natalizumab, satisfaction with frequency of out-patient appointments, experience that the physician facilitates talking about what is important for the patient, and confidence with the physician’s competence. The high overall satisfaction with health care among patients receiving natalizumab might be related to beneficial effects of natalizumab on disease activity, but might also be related to the close follow-up from specialized nurses and physicians when receiving this drug. Previous studies have also found that treatment with natalizumab have an impact on RR MS patients’ health related quality of life, regardless of disease progression [8].

As expected, satisfaction with accessibility to a physician perceived as trustworthy and qualified was associated with high overall patient satisfaction. Previous studies have also indicated that available physician services of high quality, including good communication and information, influence patient satisfaction and outcome [1, 2, 7, 12, 14, 15, 17].

Demographic factors and patient expectations did not show significant association with overall patient satisfaction. The first finding is in accordance with other studies [13, 14], whereas the latter is in contrast to other findings [12, 19]. The discrepancy may be due to inadequate interrogation of expectations in our study.

### Strength and weaknesses

A strength of this study is that all patients with a definite diagnosis of MS with follow-up at Sørlandet Hospital, Kristiansand were invited to report their view. A weakness is that only 48% of them responded. This may have led to a selection bias probably towards overestimation of patient satisfaction. Due to the anonymity of the questionnaires we could not extract further data on the profiles of the non-responders. On the other hand anonymity can be considered beneficial by warranting more honest replies from patients. The distribution of demographics and disease characteristics however, indicate that our patients were quite representative for MS patients, and that our results are applicable for relevant MS care in a general neurology out-patient setting.

An R squared in the multivariate regression analysis of 0.673 indicates that several factors not assessed in this study may influence overall patient satisfaction. Such factors may be depression, side effects, co-morbidity and functional status, or care given by other health care professions than physicians. Further, a weakness with our regression analyses is that several variables significantly associated with overall satisfaction in univariate analyses were excluded due to missing values; for example satisfaction with duration of consultations, opinion of receiving wrong treatment, and availability of health personnel.

## Conclusion

MS patients with follow-up at Sørlandet Hospital, Kristiansand reported the content of follow-up, including patient–physician interaction, as rather good. They were, however, not satisfied with the structure of the follow-up with too sporadic appointments, and several patients missed an available and named contact physician. The factors that were most associated with a high overall satisfaction with health care services were regular contact with the out-patient clinic and good quality of communication. Our results show that organizational changes are warranted to improve MS health care services. Further research should focus on how patient experiences and evaluations could be integrated in this process.

## Additional files

**Additional file 1.** A sample of the questionnaire translated from Norwegian to English by the corresponding author.

**Additional file 2.** The study’s dataset translated to English in an Excel format.

## Abbreviations

MS: multiple sclerosis
PREMs: patient reported experience measures

## References

[1] Gottberg K, Einarsson U, Ytterberg C, von Fredrikson S, Koch L, Holmqvist LW (2008). Use of health care services and satisfaction with care in people with multiple sclerosis in Stockholm County: a population-based study. Mult Scler (Houndmills, Basingstoke, England).

[2] Nylenna M, Bjertnaes ØA, Saunes IS, Lindahl AK. What is good quality of health care?. Professions and Professionalism, 2015;5(1). doi:10.7577/pp.909.

[3] Frich JC, Ramleth O (2004). Patient satisfaction as quality indicator of specialist health services. Tidsskr Nor Laegeforening Tidsskr Prakt Med Ny Raekke.

[4] Garratt A, Bjertnaes OA, Krogstad U, Gulbrandsen P (2005). The patient experiences questionnaire PasOpp in somatic outpatient clinics. Tidsskr Nor Laegeforening Tidsskr Prakt Med, Ny Raekke.

[5] Manary MP, Boulding W, Staelin R, Glickman SW (2013). The patient experience and health outcomes. N Engl J Med.

[6] Weldring T, Smith SM (2013). Patient-reported outcomes (PROs) and patient-reported outcome measures (PROMs). Health Serv Insights.

[7] Black N, Varaganum M, Hutchings A (2014). Relationship between patient reported experience (PREMs) and patient reported outcomes (PROMs) in elective surgery. BMJ Qual Saf.

[8] Miller D, Rudick RA, Hutchinson M (2010). Patient-centered outcomes: translating clinical efficacy into benefits on health-related quality of life. Neurology.

[9] Prakash B (2010). Patient satisfaction. J Cutan Aesthet Surg.

[10] Helsedirektoratet. Nasjonale faglige retningslinjer for diagnostikk, atakk-og sykdommsmodifiserende behandling av multippel sklerose. Oslo: Helsedirektoratet; 2011. p. 73s.

[11] Vatne A, Mygland A, Ljostad U (2011). Multiple sclerosis in Vest-Agder county, Norway. Acta Neurol Scand.

[12] Bjertnaes OA, Sjetne IS, Iversen HH (2012). Overall patient satisfaction with hospitals: effects of patient-reported experiences and fulfilment of expectations. BMJ Qual Saf.

[13] Pini A, Sarafis P, Malliarou M, Tsounis A, Igoumenidis M, Bamidis P (2014). Assessment of patient satisfaction of the quality of health care provided by outpatient services of an oncology hospital. Glob J Health Sci.

[14] Solari A, Martinelli V, Trojano M, Lugaresi A, Granella F, Giordano A (2010). An information aid for newly diagnosed multiple sclerosis patients improves disease knowledge and satisfaction with care. Mult Scler (Houndmills, Basingstoke, England).

[15] Ytterberg C, Johansson S, Gottberg K, Holmqvist LW, von Koch L (2008). Perceived needs and satisfaction with care in people with multiple sclerosis: a two-year prospective study. BMC Neurol.

[16] NICE. Multiple sclerosis; management of multiple sclerosis in primary and secondary care 2014 February 8th 2016.

[17] Tallantyre EC, Wardle M, Robertson NP (2016). How to run a multiple sclerosis relapse clinic. Pract Neurol.

[18] Kroll T, Neri MT (2003). Experiences with care co-ordination among people with cerebral palsy, multiple sclerosis, or spinal cord injury. Disabil Rehabil.

[19] Sorlie T, Busund R, Sexton HC, Sorlie D (2005). Patient satisfaction after hospitalisation for surgery. Tidsskr Nor Laegeforening Tidsskr Praktisk Med Ny Raekke.

