# Supplementary material for: Quality of multiple sclerosis out-patient health care services with focus on patient reported experiences
Source: BMC Res Notes. 2017 Jul 6;10:250. doi: 10.1186/s13104-017-2568-y (PMC5501112; doi:10.1186/s13104-017-2568-y)
Supplement: Supplementary file 2 — Additional file 2. The study’s dataset translated to English in an Excel format. [file 13104_2017_2568_MOESM2_ESM.docx]

Request of participation in the study

***”MS follow-up in Vest Agder – Patient satisfaction”***

**Dear MS patient**

# Background

This is a request for you with Multiple Sclerosis (MS) to participate in a research project. We want to assess how satisfied/dissatisfied MS patients are with the different aspects of the follow-up at Sørlandet Hospital, Kristiansand. Physicians at Sørlandet Hospital are performing the study.

**What happens with your information?**

The information that is registered about you will only be used as described under Background. All information will be handled anonymously. It will not be possible to identify you in the results of the study when it is published. Participation in the study is voluntary.

The study has received funding from the Norwegian MS association and has been approved by them.

**What are you going to do?**

If you don’t feel that the questionnaire is applicable to you check off the first question and return the form blank in the preaddressed envelope.

If you wish to participate in the study, fill out the questionnaire and return it in the preaddressed envelope within 14 days. If you have any questions call 38073910 and ask for one of the project managers.

Thank you!

Best regards,

Anne Marit Solheim Åse Mygland Unn Ljøstad

Physician in specialization Specialist, Professor Dr Med Specialist, PhD

**Not applicable to me**

I don’t have MS, the letter is a misunderstanding: ______

I have MS, but has follow-up somewhere other than the hospital in Kristiansand: _____

I don’t wish to participate: __________

**How satisfied are you with the help you have received for your MS disease at Kristiansand hospital?**

| Not at all | To a small extent | To some extent | To a large extent | To a very large extent |
| --- | --- | --- | --- | --- |
| □ | □ | □ | □ | □ |

**BACKGROUNDINFORMATION:**

**Age:**__________________________________________________________

**Sex:**_____________________________________________________________

**Education after elementary school:**

Three years or less: _______ Between three and six years:_______ Over six years:_______

**Work:**

Full time:____ Part time:____ Disabled :___Other (specify):________________________

**Marital status:**

Single:__________ Married/cohabitant:___________ Other:_______________________

**ABOUT YOUR MS DISEASE**

**How many years has it been since you received the diagnosis MS?** _______________________________

**What kind of MS do you have?**

Relapse-remitting: _____ Secondary progressive:______ Primary progressive:______

I don’t know: ______

**How far can you walk without aid?**

As far as I want: _______________ Over 100 m:____________ Under 100 m:______________

**Do you use aids in your daily life?**

Wheelchair: _________ Walker:____________ Cane/crutch:________ Other:___________

**What MS treatment are you receiving now?** _______________________________________

**What MS treatments have you received earlier**_____________________________________

____________________________________________________________________________

**What is your greatest symptom related to your MS?** ______________________________

**How much do you consult your GP on MS related issues?**

| Not at all | To a small extent | To some extent | To a large extent | To a very large extent |
| --- | --- | --- | --- | --- |
| □ | □ | □ | □ | □ |

**Did you have expectations of receiving good treatment at the hospital when diagnosed with MS?**

| Not at all | To a small extent | To some extent | To a large extent | To a very large extent |
| --- | --- | --- | --- | --- |
| □ | □ | □ | □ | □ |

**Do you know who your contact physician is at the neurological department at Kristiansand hospital?**

Yes:___________ No:____________

**How often do you have your regular appointments with a neurologist (not counting emergency contacts or relating to treatment with Tysabri?**

Several times a year:_____ Once a year:______ Every second year:_____ More rare:______

|  | Not at all | To a small extent | To some extent | To a large extent | To a very large extent |
| --- | --- | --- | --- | --- | --- |
| **Do you think the out-patient appointments are often enough?** | □ | □ | □ | □ | □ |
|  |  |  |  |  |  |
| **Do you think the out-patient appointments last long enough?** | □ | □ | □ | □ | □ |
|  |  |  |  |  |  |
| **Does it happen that your outpatient appointments are later than previously agreed upon?** | □ | □ | □ | □ | □ |
|  |  |  |  |  |  |
| **Have you seen many different physicians in the outpatient clinic?** | □ | □ | □ | □ | □ |
|  |  |  |  |  |  |
| **Are you satisfied with the availability of the physician?** | □ | □ | □ | □ | □ |
|  |  |  |  |  |  |
|  |  |  |  |  |  |

**Have you seen a MS nurse?**

Yes:___________ No:____________

|  | Not at all | To a small extent | To some extent | To a large extent | To a very large extent |
| --- | --- | --- | --- | --- | --- |
| **Are you satisfied with the availability of the MS nurse?** | □ | □ | □ | □ | □ |
|  |  |  |  |  |  |
| **If you have received/are receiving MS treatment (Tysabri, Novantrone, interferones or Copaxone) – are you satisfied with the follow-up (bloodsamples, MRI, physician and nurse follow-up)?** | □ | □ | □ | □ | □ |

|  | Not at all | To a small extent | To some extent | To a large extent | To a very large extent |
| --- | --- | --- | --- | --- | --- |
| **Do you trust the physician’s professional competence?** | □ | □ | □ | □ | □ |
|  |  |  |  |  |  |
| **In your opinion, have you been submitted to wrong treatment in any way?** | □ | □ | □ | □ | □ |
|  |  |  |  |  |  |
| **Do the physicians speak to you in an understandable way?** | □ | □ | □ | □ | □ |
|  |  |  |  |  |  |
| **Have you received adequate information about your disease and treatment options?** | □ | □ | □ | □ | □ |
| **Do Do think the physicians facilitate talking about what is important to you?think the physicians facilitate talking about what is important to you?** |  |  |  |  |  |
| **Do think the physicians facilitate talking about what is important to you?** | □ | □ | □ | □ | □ |
|  |  |  |  |  |  |
| **Are you involved in decisions involving your treatment?** | □ | □ | □ | □ | □ |

**Write more comments or suggestions for improvements here:**
